# Notes and News

**Published:** 1886-11-06

**Authors:** 


### NOTES AND NEWS.

Collected and Communicated.

His Royal Highness the Duke of Cambridge has intimated his intention to be present at the Conversazione of the Hospitals Association on the 17th inst., should he be in London, as he hopes may be the case.

---

H.R.H. the Duke of Cambridge is president of some of the best managed London hospitals, and has always shown the greatest personal interest in their welfare and prosperity. In these circumstances much interest will be excited by a contribution from H.R.H., on "The Importance of Maintaining the Medical Charities," which we hope to publish next week.

---

Lord Vernon has consented to act as president of the Children's Hospital at Derby for the ensuing year.

---

The lecturer on Midwifery to the female class of the Medical College of Madras is a lady, Mrs. Scharlieb, M.D., who took high honours at University College and at the London Medical School for Women.

---

At a special meeting of the Governors of the Newcastle Hospital for Sick Children on the 22nd October, Dr. Beatley was elected Physician, in place of Dr. Houseman, resigned. There were three other candidates, and the contest was a close one.

---

Mr. Thomas Lemon has been elected, out of several candidates, to the office of Apothecary at Sir Patrick Dun's Hospital in Dublin.

---

At a meeting of the subscribers to the Bridgwater Infirmary on the 22nd Oct., Mr. E. G. Broderip was appointed President for the ensuing year. He will be ably assisted in the management of the institution by Mr. John Coombs, who is again honorary secretary, and Mr. J. T. Nicholetts, the treasurer.

Mr. Henry Gilbert Nicholson, who was formerly house-surgeon at the Middlesex Hospital, and has since gained a varied medical experience both by land and sea, has recently been elected house-surgeon of Hereford General Infirmary, *vice* Mr. M. D'Oyley Gilkes, resigned. Mr. Nicholson has twice visited the West Coast of Africa in his official capacity as surgeon on board the Telegraph Construction Company's boat *Scotia*, and gained much credit for his great skill and unremitting care of the ship's company during their stay in that unhealthy climate.

---

The next monthly lecture at the Matrons' Aid Society will be given on November 5th, at 8 p.m., by Mr. Pepper, one of the surgeons at St. Mary's Hospital, and the subject will be "The Modes of Death."

---

The Scholarship of £125, open to the sons of medical men, has been awarded to Mr. A. J. Lattey, of St. George's Medical School; the Dauntsey Medical Scholarship, value about £100, to Mr. H. Horrocks, of Owens College, Manchester; the Holt Tutorial Scholarships of £100 each to Messrs. A. W. Collins and J. E. Gemmell, of University College, Liverpool; and the Charlton Scholarship, value £35, the Dickinson Scholarship of £15 and a gold medal, to Mr. B. G. Sumpter, of the University of Durham College of Medicine.

---

A verdict of manslaughter was returned by a coroner's jury on Friday against a man named Stott, an inmate of the Earlswood Asylum for Idiots, who had inflicted injuries upon another inmate named Randall, which resulted in his death. Randall, it appeared, struck the first blow.

---

There has been a serious increase in the number of fever cases in the Metropolis during the past fortnight. During that period no less than 184 patients have been received at the three principal institutions, *i.e.*, the Eastern Asylum, the Western (Fulham) Asylum, and the South Eastern Asylum, as against 173 in the preceding fortnight. On the 23rd inst. there were 622 patients in the three asylums, as against 546, the return for the fortnight ending Oct. 9th.

---

At a meeting of the Metropolitan Asylums Board on Oct. 23rd, a report was presented by Dr. Bostock, C.B., late Deputy Inspector-General, which discloses a state of affairs at the Western Asylum, which is very far from satisfactory. The recent extension of the hospital has brought the buildings over the drains, which were originally made in the open grounds, and are now stated by Messrs. Harston, the engineers commissioned to examine them, to be in a very defective condition. Indeed, the report stated that the only satisfactory and economical course will be to reconstruct the entire system. Whether or not this step will ultimately be taken is uncertain, as it was decided to consult some civil engineer well known for his experience in sanitary matters.

Dr. Geo. W. Potter and Mr. E. Austin attended at the Clifton Hall, Camden Town, on Monday evening, as a deputation from the Great Northern Central Hospital to receive the proceeds of a Trade Societies' Demonstration and Church Parade, which had been undertaken by those Societies on behalf of the building fund of that institution. There was an attendance of upwards of 500 people, chiefly of the working classes. The Rev. E. Haigh, M.A., Vicar of St. Luke's, Milner Square, presided. The treasurer of the movement handed to the deputation a cheque for £80.

---

In the course of a brief address, Dr. Potter said that the members of the Trade Societies deserved the heartiest thanks of the hospital authorities. He did not know how it was, but it seemed next to impossible to charm any money out of the tightly-buttoned pockets of the people of North London. Whether it was the incapacity of the committee, or the close-fistedness of the public, he could not tell, but there was a fault somewhere. But whether the building of the hospital proved to be easy or difficult, the committee had made up their minds that it had got to be done. The hospital was urgently needed, and by hook or by crook it must be built. Dr. Potter stated that so vast was the population, that if each grown-up person in the North of London would contribute only five shillings, the fifty thousand pounds required would be at once raised. He hoped for their own credit's sake that the inhabitants of the district would not be so utterly regardless of their duty as to leave the hospital unbuilt for want of such a comparatively insignificant sum as each individual was required to give. It would be to their lasting discredit if they did not set to work with a will and build the hospital out of hand.

---

The members of Friendly Societies all over the country would do well to follow the example of the Order of Foresters at Merthyr Tydvil, which has recently sent a subscription of £10 to the building fund of the proposed General Hospital in that town. This is a sensible and substantial recognition of the benefits conferred upon these associations by the hospitals, which not only grant care and attention to individuals who would otherwise be unable to command it, but, by their ready reception and skilful treatment of the sick, give a very real relief to the public purse of such a Society.

---

The Committee of the Dudley Dispensary report a satisfactory result from their experiment commenced in April last, when they decided to extend the issue of tickets to congregations, friendly societies, and workmen's collections. It is most desirable to encourage the masses to subscribe for the support of what are essentially working-men's institutions, and we congratulate the Committee of Dudley Dispensary on their good sense in deciding to recognise joint subscriptions in the same manner as individual donations.

---

Out of fifty-nine typhoid fever cases treated at the London Temperance Hospital during the past ten and a half years, six patients died, and they are stated to have been non-abstainers. Twenty of the fifty-three who recovered were abstainers, and thirty-three non-abstainers. No alcohol was given in any case.

---

Our contemporary, *Truth*, has again opened its annual fund for providing toys for the poor children in our metropolitan hospitals, infirmaries, and workhouses. Last Christmas over thirteen thousand children were made happy, at all events temporarily, by the generosity of the contributors to this fund, which is certainly the outcome of a very happy idea. In this department of his varied occupations, we wish the Editor of *Truth* every possible success.

---

The Governors of the Hospital for Sick Children at Sunderland have agreed to amalgamate their institution with the Infirmary, on the understanding that the new "Hartley" wing is to be devoted to the exclusive treatment of children. The cost of the new wing, including ward fittings, furniture, etc., will, it is believed, amount to £9,000, and the buildings are to be commenced as soon as £5,000 has been collected. Subscriptions to the extent of £3,200 have already been promised.

---

A Cottage Hospital nurse sends the following as "a true item of news":—

"Some time ago, a town doctor was called in to see a country patient, who was suffering greatly. Among other things the doctor ordered a light diet. On calling the next day, he asked, 'Have you obeyed my orders concerning the diet?' 'Oh, yes, doctor,' she exclaimed; 'I have had the very lightest food possible. I dined to-day off hot buttered muffins and cockles!'"

---

The following vacancies are announced this week, the amount of the salary being placed in each case after the name of the institution:—

**Honorary Medical Staff.**

Sunderland Children's Hospital, registered.—Two vacancies.

**Non-Resident Medical Officers.**

Ironmongers' Company, Fenchurch Street, E.C. Medical Officer to Almshouses, Kingsland Road, E., registered.—£52 10s.

Stockton-on-Tees Hospital, doubly qualified.—£200 per annum.

**Resident Medical Officers.**

Halifax Infirmary, doubly qualified.—£80 to £100, with board.

**District Matron.**

Glasgow Nursing Association, 2[illegible]0, Sauchiehall Street.—£60.

**Trained Nurses.**

Bolingbroke Hospital, Wandsworth Common (Lady).—£20 and uniform.

Kent and Canterbury Hospital. Surgical, night, certificated.—£22, with uniform.

Royal Hospital, Portsmouth. Male accident, night.—£24, with uniform.

Bradford Eye and Ear Hospital. Two.—£25, with uniform.

St. Giles's Workhouse, W.C. Night nurse, £20 to £26, with uniform.

**Probationers.**

Chalmers Hospital, Banff, N.B. Two. Ladies preferred.

Royal Hospital, Portsmouth. Several.—£10, laundry and uniform.

Bolingbroke Hospital, Wandsworth Common. One.

St. George's Infirmary, Fulham Road. Two. Under 25, £22 10s to £27 10s., without uniform or beer.

**Resident Clinical Assistants.**

West Riding Asylum, Wakefield. Qualified. Board and Residence.—No salary.

Mr. Easterbrook informs us that the Prince's Cinderellas, organised on behalf of the Chelsea Hospital for Women, will be resumed early in December. Some novel attractions are promised, including a Fancy Dress Ball for adults and children, with Coote and Tinney's band. Several new names will appear on the list of patronesses and stewards, who alone have the power to give introductions. Anyone interested may obtain all particulars on application to the secretary, Mr. John H. Easterbrook. The Prince's Cinderellas were such a substantial success under the late secretary, that there should be no doubt as to the result under the new management. Several hundred pounds a year is worth working for, and such a sum has been made more than once by dances on behalf of hospitals.

---

Mr. W. F. White has just completed the portrait of Mr. Francis Hawkins of Lugg Vale, Hereford, the well-known ex-Chairman of the Board of Management of Hereford Infirmary, which he has been painting for the "Hawkins Ward" of that institution. This ward owes its existence to Mr. Hawkins's princely generosity, and much of its excellence to his able suggestions and constant supervision. Hence this gift, on the part of "a few friends and admirers," will be not only an adornment of the "Hawkins Ward," but an appropriate compliment to a public benefactor.

---

Mr. Sinclair Dunn, the well-known vocalist, gave last Friday morning at Schriber's Rooms, 18, Berners Street, a highly successful concert in aid of the West-End Hospital for Diseases of the Nervous System, Paralysis, and Epilepsy. He was well supported by Miss Kate Fusselli, Miss Susetta Fenn, Miss Annie Mallows, Mr. Garry, and Mr. Ridding.

---

The Royal Infirmary of Aberdeen is one of the oldest and one of the most useful charities which Scotland boasts. We regret, therefore, to observe that the receipts have of late materially fallen off, owing, the managers believe, to the prevailing depression of trade. Last year, for instance, while the total out-going expenses amounted to £7,171 5*s.* 1*d.*, the income fell short of this amount by £956 3*s.* 2*d.* The threatened curtailment of the usefulness of the charity, in the shape of a reduction in the number of beds, will, it is earnestly to be hoped, not be found necessary. The managers have already introduced some excellent reforms, which ought certainly to work favourably in the matter of finance.

---

From the South comes the same unsatisfactory news as from the North. On the 25th ult., the Governors of the Taunton and Somerset Hospital held their annual meeting, and the report of the committee, while very encouraging in some respect, indicated that the financial condition of the institution was in anything but a satisfactory state. From 1867 to the present date, the general expenditure of the hospital has shown a pretty constant increase; but, on the other hand, the income from year to year has ebbed and flowed, till at last the tide of public charity has receded so far out to sea that the committee have grown anxious about its return. For three years the expenditure of the hospital has exceeded the income, the deficit during the past twelve months amounting to £574 15*s.* The committee attribute the diminution of the income to two causes: (*a*) the cessation of subscriptions by the death or removal of friends; and (*b*) by the exhaustion of a fund, which having been given to the hospital for the express purpose of establishing a sanatorium, or in some way supplying this want, has been for the past three years expended in sustaining three beds at the Western Sanatorium for the use of convalescents from the Taunton and Somerset Hospital. But all hospitals must suffer the annual loss of some of their old friends. If some are going, others should be coming. Surely a rich and prosperous county like Somerset should take a just pride in the efficient working of its hospital, and build up the noble work to which Dr. Malachi Blake, the generous founder, put his hand.

---

The correspondence published in the *Darlington Northern Star* of Oct. 26th, under the heading of "The Darlington Hospital Scandal," contains some very extraordinary statements. To the outside public it will seem an almost incredible thing that the committee of any hospital should have so strong "personal attachment to any matron, as to prefer parting with their *honorary* medical and surgical officers, dismissing their house-surgeon, discharging their efficient trained nurses, and accepting the resignations of three members of their body, to sending away one who seems to consider her high official position only as an efficient instrument for insulting gratuitously, or otherwise, all whose work brings them into contact with her." One resigning member of the committee, in his explanatory letter, states that a "faithful, laborious, and skilful house-surgeon" was summarily dismissed, "for the sake of one against whom complaints came in showers, and never ceased." All this is greatly exaggerating an unfortunate quarrel which ought never to have occurred, and which is now happily in a fair way of being settled. We have ascertained that the public scepticism is justified by the facts, and that much of the heat generated has been due to the indiscretion and letter writing of the resigning members of the committee, and to the editor of the *Northern Star*. There is an old and useful proverb about dirty linen and home, which both would have done well to remember.

---

As we go to press the Governors of the Newcastle Infirmary are considering a question of the very gravest interest. For some years past this institution has been drawing on its funded capital. At present the deficiency to be met amounts to about £5,000 per annum, and as the capital has already been reduced to £45,175, it is obvious that a few years of such necessity would, unless a change be made, seal the fate of this admirable institution. The proposition which the Governors will discuss is one for curtailing the expenditure by reducing the average number of patients by 70. Such a decision, if come to, would stultify the good work of the Newcastle Infirmary, without materially reducing the expenditure, and it is to be earnestly hoped that the generous townsfolk will come to the assistance of their institution in its hour of need.

